# Integration of PK/PD for dose optimization of Cefquinome against *Staphylococcus aureus* causing septicemia in cattle

**DOI:** 10.3389/fmicb.2015.00588

**Published:** 2015-06-17

**Authors:** Ijaz Ahmad, Haihong Hao, Lingli Huang, Pascal Sanders, Xu Wang, Dongmei Chen, Yanfei Tao, Shuyu Xie, Kuang Xiuhua, Juan Li, Wan Dan, Zonghui Yuan

**Affiliations:** ^1^National Reference Laboratory of Veterinary Drug Residues and MAO Key Laboratory for Drug Residues Detection, Huazhong Agriculture UniversityWuhan, China; ^2^MOA Laboratory for Risk Assessment of Safety and Quality of Livestock and Poultry Products, Huazhong Agriculture UniversityWuhan, China; ^3^Hubei Collaborative Innovation Centre for feed Safety and Animal Nutrition, Huazhong Agricultural UniversityWuhan, China; ^4^Laboratory of Fougères, French Agency for Food, Environmental and Occupational SafetyMaisons-Alfort, France

**Keywords:** cefquinome, *Staphylococcus aureus*, cattle, PK/PD, septicemia

## Abstract

Cefquinome is a fourth generation cephalosporin with antimicrobial activity against gram negative and gram positive bacterial species, including *Staphylococcus aureus*. The aim of our study was to observe the *ex-vivo* activity of cefquinome against *Staphylococcus aureus* strains by using bovine serum from intravenously treated cattle. Cefquinome kinetics were measured by liquid chromatography and UV detection. *In vitro* post antibiotic effects (PAEs) and mutant prevention concentrations were determined with *S. aureus* strain ATCC 12598. Cefquinome exhibited time-dependent killing and produced *in vitro* PAEs increasing with concentration and time of exposure. A pharmacokinetic-pharmacodynamic model was established to simulate the efficacy of cefquinome for different dosage regimens. A dosage of 2 mg/kg every 12 h for 3 days was expected to reach a bactericidal activity against *S. aureus* in case of septicemia.

## Introduction

*Staphylococcus aureus* is a gram positive bacteria, responsible for many animal and human diseases. It can cause an extensive variety of infections from skin and soft tissue infections to septicemia. If it is not treated efficiently, the sepsis can produce inflammatory response, organ dysfunction syndrome, shock, and finally death (Fecteau et al., 2009; Fluit, 2012). During the first day of life septicemia commonly occurs in calves. Bacteremia was observed during the neonatal period in 20–30% of diarrheic calves. In bacteremia or septicemia, the bacteria isolated included *Escherichia coli*, *Salmonella* spp, *Campylobacter* spp, *Klebsiella* spp., and *Staphylococcus* spp (Raboisson et al., 2010). Consequently, *S. aureus* has a documented effect on mortality, with related rate of mortality at 20–40% (Uramatsu et al., 2010). Since, staphylococcal diseases are usually treated with antibiotics there is an associated risk to select antibiotic resistance (Normand et al., 2000). Without treatment, the mortality rate will be high (Gordon, 1998).

Rational Antibiotic therapy needs to ensure clinical efficacy and to reduce the risk of antibiotic resistance selection and amplification (Toutain et al., 2002; Ambrose et al., 2007; Jacobs, 2007; Tam et al., 2007). The beta-lactam antiobiotic is safer in the target animals as compared to other antibiotics (Dumka et al., 2013). The beta lactam antibiotic is time dependent and its bactericidal activity is lower as compared to other bactericidal antibiotics. Also, it has no or a minimal post antibiotic effect. Therefore to optimize the efficacy, the drug concentration should be maintained above the MIC for a longer time during the dosing interval at the site of infection (McKellar et al., 2004; Owens and Ambrose, 2007; Zonca et al., 2011; Papich, 2014). Cefquinome is the fourth generation cephalosporin, mainly used in veterinary medicine. This drug has been developed especially for use in veterinary medicine (Smiet et al., 2012; Papich, 2014), and is registered in many countries worldwide. Cefquinome has been used for the treatment of many diseases including acute mastitis, respiratory diseases, food rot in cattle, calf septicemia, metritis-mastitits-agalactia syndrome in sows, foal septicemia and respiratory diseases in horses (Uney et al., 2011; Liu et al., 2012; Dumka et al., 2013; Shan et al., 2014). The advantages of cefquinome include broad spectrum antibacterial activity, stability against β-lactamase, enhanced potency, and bioavailability and the ability to penetrate easily into gram negative bacteria (Dumka et al., 2013). Cefquinome pharmacokinetics (PK) have been studied in several animal species such as camels, horses, ducks, cows, wild boars, piglet, rabbits and cattle (Allan and Thomas, 2003; Ehinger et al., 2006; Li et al., 2008; Al-Taher, 2010; Hwang et al., 2011; Liguo et al., 2011; Liu et al., 2012; Shan et al., 2014).

In human medicine, international guidelines for management of severe sepsis underline the needs to base the choice of antibiotics on the knowledge of their pharmacokinetics and their pharmacodynamics (PK/PD) (Dellinger et al., 2008; Solomkin et al., 2010; Duszyńska, 2012). The use of PK/PD modeling has also become common in the veterinary literature. PK-PD principles are an important tool to be used by the regulatory authority when studying the application of an antimicrobial. It was also stated that the single most important factor responsible for the emergence of resistance is bacterial exposure to sub optimal concentration of an antibiotic (Papich, 2014). Therefore, some strategies have been established for dosage regimens to attain appropriate PK/PD targets in severe infection and to minimize selection of antibiotic resistance (Drusano, 2004; Olofsson and Cars, 2007; Duszyńska, 2012). Recently, one researcher investigated the pharmacokinetics and *ex-vivo* pharmacodynamics activity of cefquinome by using tissue cage fluid and serum obtained from pigs (Zhang et al., 2014). Efficacy of different dosage regimens was investigated in a *Staphylococcus aureus* infection in a thigh model of neutropenic mouse (Wang et al., 2014).

The objectives of the present study were to examine the PK and, establish *in vitro* PD parameters and *ex-vivo* PD characteristics of cefquinome in serum for integration in a PK-PD model for cefquinome for treatment of *Staphylococcus aureus* systemic infections in calves.

## Materials and methods

### Animals

All the experimental procedure in this study were performed according to the guidelines of the committee on the use and care of the laboratory animals in Hubei province China. The study was approved by the Animal Care Center, Hubei Science and Technology Agency in China (SYXK 2013–0044). All the animals were monitored throughout the study for any adverse effect signs. The study was conducted in 6 healthy cattle calves of 6–10 months of age. Body weights (BW) was 185 ± 10 kg. They were housed in a 8 × 10 m cattle pen, and the pen was cleaned daily. The room temperature was 25 ± 2°C and a relative humidity of 45 65% was maintained in the animal house. All animals were allowed a 15 days acclimation period before the study began. The water and feed for the animals was available *ad libitum*. None of the animals had been treated with antibiotics before.

### Drug administration and sample collection

Each calf received cefquinome (Cefquinome sulfate 2.5%, Shanghai Tongren Pharmaceutical Company Ltd.) at a dosage of 1 mg/kg BW. The drug was administered intravenously to 6 cattle by jugular vein at the dose rate of 1 mg/kg. The blood samples (5 mL) were collected before and at 0.25, 0.5, 1, 2, 3, 4, 6, 8, 12, and 24 h after cefquinome administration from opposite jugular vein. Blood samples were collected into EDTA tubes and tubes without anticoagulant. Plasma and serum were obtained immediately by centrifugation at 3000 *g* for 20 min at 4°C and the supernatants (plasma or serum) were stored at −20°C until analysis.

### Drug analysis

#### Chemicals and reagents

The cefquinome reference standard (95% purity) was kindly supplied by Dr. Ehrenstorfer (Augsburg, Germany). The reagents used were of analytical grade. Formic acid, methanol (MeOH), and acetonitrile (ACN) were provided by TEDIA (USA). Solid phase extraction (SPE) cartridges (Waters Oasis™ HLB, waters) were used in the analytical method. De-ionized water (Milli-Q Millipore Corp.) was used during the study.

#### Development of HPLC method

Plasma samples were analyzed using a Waters 2695 series high performance liquid chromatography (HPLC) and a Waters 2587 UV detector set at a wavelength of 268 nm. The chromatographic separation was achieved with an analytical ZORBAX SB-C_18 column_ (250 × 4.6 mm i.d., 5 μm; Agilent Technology, USA) at 30 ± 5°C. The separation was on isocratic mode with mobile phase A containing 0.1% formic acid and B acetonitrile (90/10, v/v) at 0.9 ml/min flow rate. The injection volume was 50 μL.

After thawing at room temperature, aliquots of 250 μL cattle plasma were collected in 1.5 mL tubes. Then, 500 μL methanol was added, tubes were shaken for 20 s, and were centrifuged (8000 *g*) for 10 min at 4°C. After centrifugation, the supernatant was pipetted into a tube and 10 mL water was added. The mixture was then cleaned up on a HLB SPE cartridge (3 mL, Waters Corp., Milford, MA, USA), preconditioned with 3 mL methanol and 3 mL water. After transfer, the cartridge was washed with 3 mL water and 3 mL (10% methanol). The analytes were eluted with 3 mL acetonitrile. The samples were dried with a stream of nitrogen at 50°C. The residue remaining after evaporation with nitrogen was reconstituted with 500 μL of 15% acetonitrile. After Vortex mixing for 20 s, the samples were filtered through 0.22 μm nylon Millipore chromatographic syringe filter into an autosampler vial.

For calibration, 250 μL blank plasma were spiked with 0.01–5 μL of a series of diluted cefquinome working standard solutions and analyzed as above. The cefquinome concentrations in the prepared standard samples were 0.01, 0.02, 0.04, 0.08, 0.16, 1, 2, and 5 μg/mL. Spiked quality control samples were prepared at the concentrations of 0.04, 0.08, and 0.16 μg/mL. Retention time for cefquinome in plasma was 13.40 min. The limit of detection (LOD) and limit of quantification (LOQ) were 0.01 and 0.04 μg/mL. Cefquinome quantification is linear within a range of 0.01–5 μg/mL (r^2^ > 0.999). The recovery of cefquinome from plasma was 76.24 ± 2.55% (mean ± SD). The coefficient of variability (CV %) was all <15% for intra- and inter-day variation.

#### Pharmacokinetic analysis

Pharmacokinetic analysis was performed using Win-Nonlin (version 5.2.1, Pharsight Corporation, Mountain View, CA, USA). The data obtained after plasma concentration determination were analyzed by compartmental methods. The compartmental analysis was evaluated based on Akaike Information Criteria estimates and coefficient of determination application for the best fit model (Liu et al., 2012). The data were analyzed by non-compartment modeling. To find the values of the area under the concentration-time curve (AUC) and area under the first moment curve (AUMC), we used the linear trapezoidal route. The apparent volume of distribution at steady state (VSS) and volume of distribution (VDarea) were calculated according the following equation.

$$VD_{area}=\frac{Dose}{AUC\times \beta }$$

$$VSS=\frac{Dose/AUC}{AUMC\text{ /}AUC}$$

The pharmacokinetic parameters are presented as mean ± SD.

### Pharmacodynamics

#### Bacterial strain and animals

*Staphylococcus aureus* Rosenbach (ATCC BAA-934), subsp. Aureus (ATCC-12598), and ATCC-29213 strains were purchased from the American Type Culture Collection (ATCC). *Staphylococcus aureus* subsp. Aureus Rosenbach (ATCC-12598) was isolated from septic arthritis (www.atcc.org). Thirty *S. aureus* strains isolated from healthy cattle in Huazhong Agriculture University Wuhan, China were also evaluated. The strains were stored at −80°C. Prior to each experiment, the bacteria were grown freshly on Chrom agar and MH agar and incubated at 37°C.

#### Determination of minimal inhibitory concentration, minimal bactericidal concentration, mutant prevention concentration, and post antibiotic effect

The minimal inhibitory concentration (MIC) of cefquinome against *S. aureus* strains were determined in both broth and serum by micro dilution method according to the CLSI (Clinical and Laboratory standards Institute, 2008), at concentrations between 8 and 0.015 μg/mL. Microplates were incubated at 37°C for 24 h. MIC was determined as the lowest cefquinome concentration where at the end of the incubation period for 24 h, growth of visible bacteria was inhibited. For the minimal bactericidal concentration (MBC) of cefquinome against *S. aureus* strains, 100 μL from each well were successively diluted in 0.85% sodium chloride solution by 1:10 steps and 10 μL were spread on MH agar plates for colony forming unit (cfu) counting and incubated at 37°C for 24 h. MBC was defined as the lowest drug concentration which resulted in a 99.9% reduction in the bacterial density. MBC is the result of five independent experiments. The mean was expressed as the final result.

The mutant prevention concentration (MPC) of cefquinome is determined by agar method. The inoculum of *Staphylococcus aureus* was concentrated to 10^10^ CFU/mL according (Balaje et al., 2013). Bacterial suspensions were inoculated on the agar plates containing serial dilutions of cefquinome and cultured for 96 h. MPC was the lowest drug concentration on agar plates without bacterial growth under anaerobic conditions. Drug range tested for MPC was 1 MIC, 2 MIC, 4 MIC, 8 MIC, 16 MIC, and 32 MIC.

Post-antibiotic effect (PAE) of cefquinome against *Staphylococcus aureus* is estimated with removal of drug methods. The strain of *S. aureus* was incubated with 1 MIC, 2 MIC, 4 MIC of drug. After one and two hours' incubation, the drug was eliminated by several times of centrifuge and wash with fresh medium. The colony forming units (CFU) per milliliter were determined at different time points. The recovery growth kinetic curves of bacteria were established in order to calculate the PAE.

#### *In vitro* and *ex-vivo* bacterial killing curves

After MIC and MBC determinations, different concentration of cefquinome were prepared in MHB ranging from 1/8 to 16 × MIC before bacterial inoculation (10^6^ cfu/mL). Growth was checked with a control. The tubes containing bacteria and different concentration of cefquinome were incubated at 37°C and the viable counts of bacteria were determined at 2, 5, 8, and 24 h. At each time, 100 μL obtained were sampled, gradient diluted by saline and then colony forming units were counted. The limit of detection was 10 cfu/mL.

Serum samples obtained from calves that had received cefquinome intravenously were used. Controls were prepared from serum samples collected from the same calves before administration. A 10 μL volume of bacterial culture in stationary phase was added to 1 mL of serum to give a final suspension of approximately 10^6^ cfu/mL. The tubes containing bacterial culture and serum were than incubated at 37°C, and viable counts were determined at 0, 2, 5, 8, and 24 h (Shan et al., 2014).

The time-kill curves obtained with serum were analyzed with a pharmacodynamic model described by the following equation

$$\frac{dB}{dt}=k_{net}\times \left(1-\frac{B}{B_{max}}\right)\times B-\left(\frac{E_{max}+C^{\gamma }}{EC_{50}^{\gamma }+C^{\gamma }}\right)\times B$$

Where *B*, is the number of bacterial cell expressed as cfu/mL, *k_net_* the net growth rate, *B_max_* the maximum number of bacteria, *E_max_* the maximum killing rate, EC_50_, the concentration to reach half of maximal killing rate and γ, the steepness.

The *ex-vivo* time kill curve was fitted with this model with the hypothesis of a decrease in cefquinome concentration according the incubation time using the proc lsqnonlin (Matlab).

#### Pharmacodynamic analysis, PK-PD integration and PK-PD modeling analysis

For cefquinome, the surrogate markers of antimicrobial activity, AUC_24 h_/MIC, C_max_/MIC, and T>MIC, were determined by linking *in vitro* data and *in vivo* pharmacokinetic parameters for serum.

$$\begin{array}{l} \frac{dB}{dt}=k_{net}\times \left(1-\frac{B}{B_{max}}\right)\times B-k_{death}\times B\\ \;-\left(\frac{E_{max}+C^{\gamma }}{EC_{50}^{\gamma }+C^{\gamma }}\right)\times B \end{array}$$

The relationship between the *ex-vivo* AUC_24 h_/MIC ratio and thevariation between the initial bacterial count and the bacterial count after 24 h of incubation (cfu/mL) in serum content was established by using the inhibitory sigmoid *E_max_* model (FS and Lees, 2001). This model is described by the following equation

$$E=E_{max}-\frac{(E_{max}-E_{0})\;\cdot \;C^{N}}{C^{N}+EC_{50}^{N}}$$

where *E* is the effect of antibacterial agent measured as the change in log_10_ difference of bacterial count after 24 h incubation compared with the initial log cfu/mL in the serum sample; *E_0_* is the change in log_10_ difference in bacterial count of control sample between 0 and 24 h; *E_max_* is the change in log_10_ difference in bacterial count between 0 and 24 h in the cefquinome containing samples; *EC_50_* is the AUC_24h_/MIC value producing 50% of the *E_max_*; C is the AUC_24 h_/MIC ratio being examined; and N is the Hill coefficient that describes the steepness of the (AUC_24 h_/MIC) effect curve. These PD parameters were calculated using the GraphPad Prism software (version 5.01, USA).

The *ex-vivo* antibacterial effect of cefquinome after intravenous administration were quantified MIC from the sigmoid *E_max_* equation by determining AUC_24 h_/MIC for four levels of effect: for bacteriostatic action (no change in bacterial count, that is *E* = 0), for 50% reduction in the bacterial count, for bactericidal action (a 99.9% reduction in bacterial count) and for bacterial elimination (a 99.99% reduction) (FS and Lees, 2001). The dose was calculated by the using the following formula.

$$Dose=\frac{\left(AUC_{24}\;/MIC\right)\;\cdot \;MIC\;\cdot \;CL}{fu\;\cdot \;F}$$

Cefquinome is time dependent drug and the PK/PD index responsible for the efficacy is T > *MIC*. To find *T* > *MIC*% we used the following formula

$$\text{T}≻\text{MIC}=\text{In}\left(\frac{\text{D}}{\text{Vd · MIC}}\right)\;\cdot \;\frac{\text{T}1/2\beta }{\text{In2}}\;\cdot \;\frac{100}{\text{t}}$$

Where *T* > *MIC* (in percent) is the time interval during which the drug plasma concentration is above or equal to the minimal inhibitory concentration (MIC) values; D is the planned dose; t_1/2_ the terminal elimination half-life; and t is the dose interval (Smiet et al., 2012).

To investigate the effect of different dosage regimens, the pharmacodynamic model describing bacterial growth rate in function of cefquinome concentration was combined with the pharmacokinetic model and simulations were performed with mlxplore software (version-1.1.0, Lixoft, Orsay, France).

## Results

### Pharmacokinetics of cefquinome

**Figure 1:**
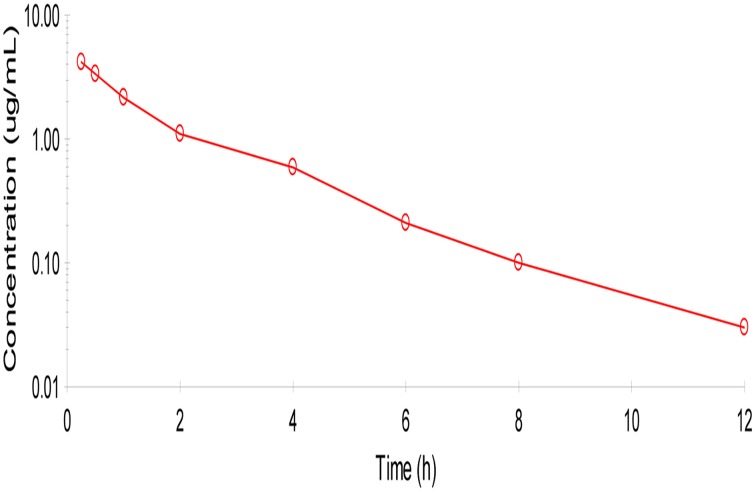
**Semi-logarithmic plot of mean serum concentration after intra venous administration of cefquinome (1 mg/kg) in cattle (*****n*** **= 6)**. Two compartment method (Winonlin software) were used to observe the concentration-time curve.

No adverse reactions were observed after intravenous drug administration. The plasma concentration-time profiles are illustrated in (Figure 1). After intravenous administration, plasma concentration of cefquinome was best fitted with a two-compartment model (Table 1). The elimination half-life was 2.1 ± 0.45 h showing rapid elimination after intravenous administration. The area under the concentration time curve AUC_0−∞_ was 8.04 ± 0.34 μg.h/mL. The volume of distribution at steady state (Vss: 0.28 ± 0.02 L/kg) was low. The mean residence time up to last was 2.3 ± 0.3 h.

**Table 1:** **Pharmacokinetics parameters after intravenous administration of Cefquinome in cattle at the dose (1 mg/kg) body weight**.

**PK parameters**	**Mean**	**SD**
AUC (μg.h/mL)	8.04	0.34
K_10_ (l/h)	0.67	0.03
K_12_ (1/h)	0.39	0.09
K_21_ (1/h)	0.69	0.24
T ½α (h)	0.48	0.09
T 1/2 β (h)	2.10	0.45
V1 (L/kg)	0.18	0.00
CL (L/h.kg)	0.12	0.00
Vss (L/kg)	0.28	0.02
MRT (h)	2.30	0.30
AUMC (μg.h2/Ml)	18.56	2.19

*AUC, Area under the concentration-time curve; K_10_, First order elimination rate constant; K_12_, First order transfer rate constant describing distribution between central and peripheral compartment; K_21_, First order transfer rate constant describing distribution between peripheral and central compartment; T 1/2α, Half-life of distribution; T 1/2β, Half-life of elimination; CL, Systemic clearance; Vss, Volume of distribution at steady state; MRT, Mean residence time; AUMC, Area under the first moment-time curve*.

### Pharmacodynamics

#### MIC, MBC, MPC, and PAE of cefquinome against staphylococcus *aureus* strains

The MIC and MBC of cefquinome against *S. aureus* were for the three tested strains at 0.25 and 0.5 μg/mL in culture medium MHB and in serum (Table 2). MIC_90_ of 30 strains isolated from healthy cattle in our laboratory was 0.25 μg/mL. The mutant prevention concentration (MPC) of cefquinome against the 3 strains in MHB and serum were 2 μg/mL.

**Table 2:** **MIC, MBC and MPC (μg/ml) of Cefquinome against** ***staphylococcus aureus***.

**Parameters**	**Matrix**	**Strain 1 (ATCC-29213)**	**Strain 2 (ATCC-BAA-934)**	**Strain 3 (ATCC-12598)**	**Mean ± SD**
MIC	MHB	0.25	0.25	0.25	0.25 ± 00
	Serum	0.25	0.25	0.25	0.25 ± 00
MBC	MHB	0.5	0.5	0.5	0.50 ± 00
	Serum	0.5	0.5	0.5	0.50 ± 00
MBC/MIC	MHB	2	2	2	2 ± 00
	Serum	2	2	2	2 ± 00
MPC	MHB	4	2	2	2.66 ± 1.15
MPC/MIC	MHB	16	8	8	10.66 ± 4.61

*MIC, Minimum inhibitory concentration; MBC, Minimum bactericidal concentration; MPC, Mutant prevention concentration*.

Post-antibiotic effect (PAE) of cefquinome for different concentrations (1x, 2x, 4x MIC) and time exposure (1, 2 h) is lower than 1 h and increases with time and concentration (Table 3).

**Table 3:** **Post antibiotic effect (PAE) after 1 and 2 h. (ATCC-12598)**.

**Antibacterial concentration**	**PAE after 1 h (h)**	**PAE after 2 h (h)**
1MIC	0.10	0.10
2MIC	0.20	0.20
4MIC	0.30	0.60

#### *In vitro* and *ex-vivo* antimicrobial activity

**Figure 2:**
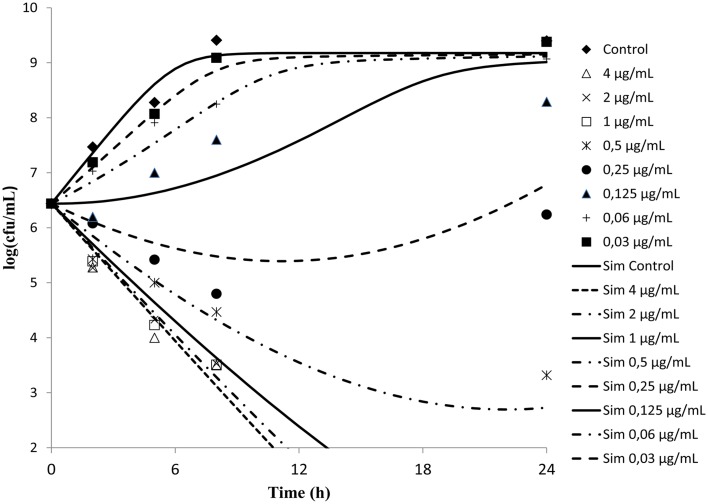
***In vitro*** **antibacterial activity of cefquinome against** ***Staphylococcus aureus*** **in MH broth**. The figures show that cefquinome exhibit time dependent activity when the concentration increases.

Time kill curves obtained against *S. aureus* ATCC-12598 for different concentrations of cefquinome expressed as multiple of MIC were reported (Figure 2). The curves were characteristics of time-dependent antibiotic activity. The net growth rate is lower for concentration below the MIC. Meanwhile the bactericidal activity increased with increasing concentration of the cefquinome up to 4 MIC. A further increase in concentration resulted in the same death rate.

**Figure 3:**
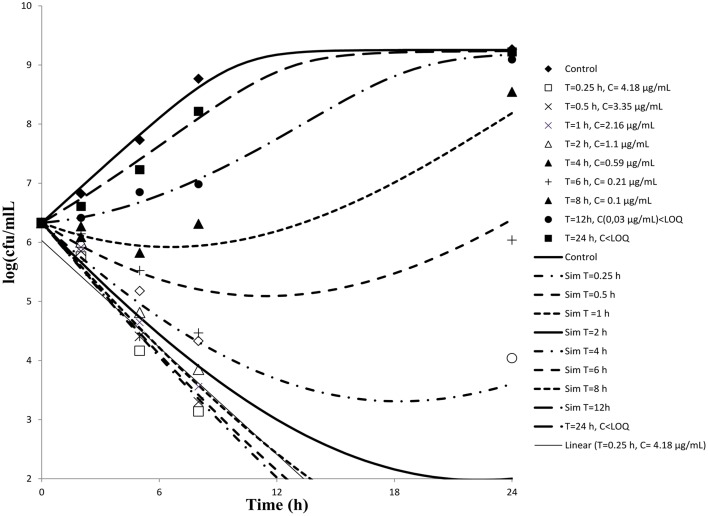
***Ex-vivo*** **antibacterial activity of cefquinome in serum of cattle against** ***staphylococcus aureus*** **after intravenous administration (*****n*** **= 6)**. The concentration of cefquinome in serum at different time interval shows its effect on *staphylococcus aureus*.

Serum samples from six cattle that had been administered cefquinome intravenously collected at different time points were used to determine *ex-vivo* killing rate. The results show that at the highest concentration, the number of bacteria decreased slightly (Figure 3). A net killing rate is obtained with samples collected before 6 h and growth is observed for serum samples collected after 8 h. In the data set with serum, a phenomenon of decrease of bacteria number followed by a regrowth was observed on the time kill curves obtained with serum sampled at 8 and 12 h. After fitting, the net killing rate was 0.95 h^−1^, the net growth rate was *k_net_* = 0.6915 h^−1^, the maximum number of bacteria *B_max_* was (10E9.25), the maximum killing rate was *E_max_* = 1.67 h^−1^, *EC*_50_ = 0.06 μg/mL, γ = 0.70 and cefquinome decrease rate = 0.15 h^−1^.

#### PK/PD integration

The mean values of the PK/PD indices C_max_/MIC, AUC_24_/MIC, C_max_/MBC, AUC_24_/MBC, and C_max_/MPC, AUC_24_/MPC of cefquinome against *staphylococcus aureus* are shown in (Table 4). The values determined for C_max_/MIC and AUC_24_/MIC were 16.72 and 32.16 h. The mean values for C_max_/MPC and AUC_24_/MPC were 2.09 and 4.02.

**Table 4:** **Integration of PK/PD data obtained after intra venous administration of Cefquinome (1 mg/kg) in cattle (*****n*** **= 6)**.

**Parameter**	**Unit**	**Mean**
C_MAX_	μg/ml	4.18
AUC_0−24 h_	μg.h/ml	8.04
MIC	μg/ml	0.25
MBC	μg/ml	0.50
MPC	μg/ml	2.00
C_MAX_/MIC	–	16.72
AUC_0−24 h_/MIC	h	32.16
C_MAX_/MBC	–	8.36
AUC_0−24 h_/MBC	H	16.08
C_MAX_/MPC	–	2.09
AUC_0−24 h_/MPC	h	4.02

#### PK/PD modeling

The association between AUC_0−24_/MIC ratio and antibiotic efficacy was best described by using inhibitory sigmoid E_max_ model. The parameters obtained N, E_0_, E_max_ and AUC_0−24_/MIC values required for various degrees of antibacterial activity are shown in Table 5. The values of AUC_0−24_/MIC ratios for bacteriostasis activity, bactericidal action and virtual eradication were 29.71, 51.97, and 67.51, respectively.

**Table 5:** **PK/PD Modeling of** ***ex-vivo*** **data after administration of Cefquinome in cattle (*****n*** **= 6)**.

**Parameters**	**Unit**	**Mean**
E_0_	Log_10_ CFU/ml	2.93
E_MAX_	Log_10_ CFU/ml	−5.33
E_MAX_-E_0_	Log_10_ CFU/ml	8.26
Slope (N)	–	2.74
AUC_24 h_/MIC EC50	h	36.96
AUC_24 h_/MIC for bacteriostatic action	h	29.71
AUC_24 h_/MIC for bactericidal action (99.9% reduction)	h	51.97
AUC_24 h_/MIC for bacterial eradication (99.99% reduction)	h	67.51

*E_0_, difference in bacterial count in control sample (without drug) between 0 and 24 h; E_max_, difference in bacterial count in sample incubated with Cefquinome between 0 and 24 h; AUC_24_h/MIC EC_50_:AUC_24_h/MIC of drug producing 50% of maximal antibacterial effect; N, slope of the AUC_24_h/MIC–response curve*.

#### Estimation of dose

The dose of cefquinome in cattle calves after a single intravenous administration, based on the observed AUC_24 h_/MIC values by modeling PK/PD data, MIC values and PAE values of our study and MIC_90_ required for bacteriostatic, bactericidal and bacterial eradication responses are 3.6, 6.4, and 8.3 mg/kg for bacteriostatic, bactericidal action and virtual eradication activity for 24 h dosage interval. The t > MIC for the above mentioned dose are 50, 57, and 60%.

**Figure 4:**
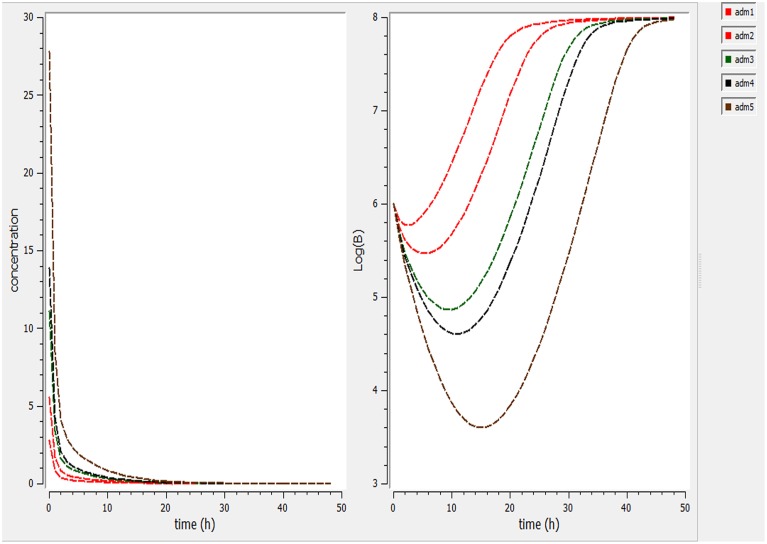
**Simulate the effect of different doses (0.5, 1, 2.5, 5, 7.5 mg/kg)**. The effect of different doses were observed on bacteria and its elimination.

Using a PK/PD model with the PD parameters derived from *ex-vivo* analysis, actions of different doses (0.5, 1, 2.5, 5, 7.5 mg/kg) were simulated (Figure 4). According these figures, a dose of 1 mg/kg is not sufficient to reduce the bacterial load while a dose of 2 mg/kg lead to a net reduction of a factor of 10 after 12 h.

**Figure 5:**
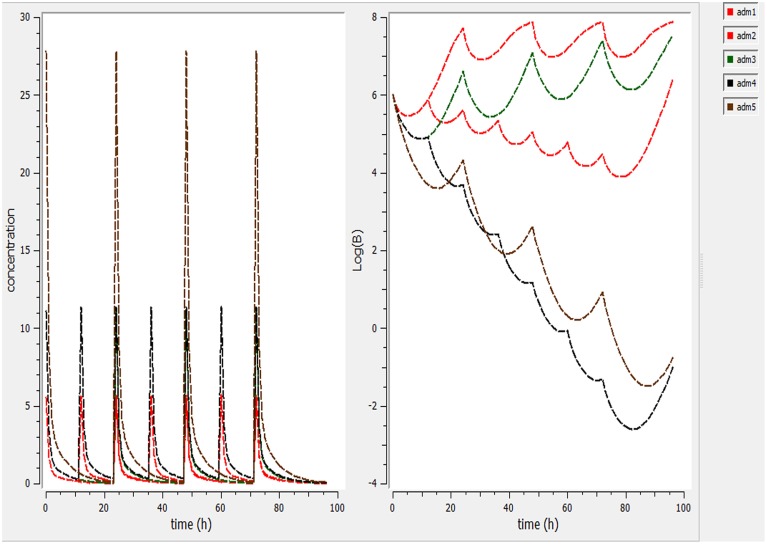
**Simulate different dosage regimen (1 mg/kg every 12 h, 1 mg/kg every 24 h, and 2 mg/kg every 24 h, 2 mg/kg every 12 h, and 5 mg/kg every 24 h)**. The different doses were simulated for different intervals of time to find the efficient dose and dose intervals

Different dosage regimen for 3 days of treatment (1 mg/kg every 12 h, 1 mg/kg every 24 h and 2 mg/kg every 24 h, 2 mg/kg every 12 h, and 5 mg/kg every 24 h) (Figure 5) were simulated. A dosage regimen of 2 mg/kg every 12 h should be efficient to reach a bactericidal activity in serum (Figure 5).

## Discussion

Pharmacokinetic properties of cefquinome have been studied previously in the serum of cattle after intravenous administration at 1 mg/kg (Shan et al., 2014). The reported terminal half life, clearance and Vss were respectively 2.4 ± 0.21 h, 0.11 ± 0.03 L/h.kg, 0.3 ± 0.5 L/kg and were very close to our values. The terminal elimination half-life of cefquinome is similar to values that were observed in piglet (Zhang et al., 2014), ducks (Liguo et al., 2011), rabbit (Hwang et al., 2011), and horses (Winther et al., 2011) in the range of (0.9–2.77 h) following IV administration. The volume of distribution at steady state was low which means that cefquinome was not as widely distributed as previously reported for piglet (Zhang et al., 2014), sheep (Uney et al., 2011), rabbits (Hwang et al., 2011), and horses (Winther et al., 2011) in the range (0.19–0.36 L/kg).

The MIC of cefquinome against *S. aureus* strains tested (0.25 μg/mL) were in the same range as those previously reported (Limbert et al., 1991; Wang et al., 2014). The MBC was determined to be two fold higher.

Cefquinome is considered to be a time-dependent agent and its activity is a function of the time remaining in excess of MIC. There was a regrowth observed in the inoculated bacteria on the *ex-vivo* time kill curves obtained for low concentration. A model with a decreasing of cefquinome concentration in serum was used to fit the data. This mathematical model is the simplest to describe a bacterial regrowth. As antimicrobial concentration in serum after 24 h of incubation was not measured, it was not supported by any data and must be considered as theoretical. A theoretical MIC value for the strain tested was calculated as 0.064 μg/mL. This value means a better susceptibility of *S. aureus* in serum than those observed after 24 h of culture in Mueller Hinton.

The PAE observed *in vitro* below 1 h is short in comparison of 2.9 h obtained *in vivo* in neutropenic mice thigh model of *staphylococcus aureus* (Wang et al., 2014). The *in vivo* PAE of cephalosporin were between 2 and 6 h in the former model. Then, PAE determined *in vitro* is a poor predictor of those observed *in vivo*. For our simulation, PAE will not be considered as our data are not enough to build a model to predict *in vivo* scenario.

The bactericidal action of a time dependent drug is relatively slow as compared to concentration dependent drugs (Levison, 2004), and little increase in bactericidal activity is seen when the concentration is increased more than maximal killing concentration, which is approximately equal to 4 times the MIC. These drugs have short or no PAE for gram negative bacilli and have short PAE for gram positive bacilli; the drug concentration above the MIC relative to dosing interval is important, consequently the dosing administration frequency is an important factor for the efficacy. Shorter dosing intervals will increase the duration of time at which the drug concentration is above the MIC of the causative agent (McKellar et al., 2004). For time dependent drugs, AUC/MIC is a poor predictor of efficacy while T > MIC is the best surrogate. To estimate this surrogate from PK and *in vitro* and *ex-vivo* data, it is necessary to estimate the maximum kill rate, EC_50_ and steepness from the time kill curve data using a differential model (Nielsen and Friberg, 2013). So use of mathematical model describing simultaneously the pharmacokinetics of a drug and the effect on bacterial growth is the best approach to investigate time dependent drugs.

The mutant selection window hypothesis was initially proposed using agar plate assays and then explored in several *in vitro* and *in vivo* models mainly for fluoroquinolones for which resistance appears by mutation. For time-dependent drugs such as cephalosporin, it was rarely reported and as far as we know, only one recent paper investigated *in vivo* the mutant selection window for cefquinome against *E. coli* in an *in vitro* model (Zhang et al., 2014). For *S. aureus*, the observed *in vitro* MPC (2 μg/mL) was 8 times higher than the MIC (0.25 μg/mL). Compared with the MIC definition, the mutant prevention concentration (MPC) is defined as the lowest drug concentration that prevents the growth of the least susceptible first-step resistant mutants. It has been proposed that the AUC_24 h_/MPC ratio could help as an indicator of drug exposure that stops the selection of drug-resistant mutants (Zhao and Drlica, 2001; Olofsson et al., 2006). Further investigations are needed to determine the mechanisms of resistance (induced or acquired) observed in our study. For this reason, mutant prevention concentration was not taken into account in our dosage regimen optimization.

PK/PD model helps as a bridge between *in vitro* and *in vivo* study and permits predicting the suitable dose for bacteriological bend and emergence. According our pharmacokinetic and pharmacodynamic parameters, single dose to reach bacteriostatic, bactericidal and eradication activity corresponding to 3.6, 6.4, and 8.3 mg/kg doses to maintain a concentration higher than the observed MIC (0.25 μg/mL) for a fraction of 50, 57, or 60% of a 24 h interval (Figure 4). For the treatment, we simulate different dosage regimen and show that a 3 day treatment of 2 mg/kg every 12 h should be efficient against *S. aureus* ATCC 12598 in cattle. These PK/PD interrelationships expect that cefquinome treatment is likely to be effective clinically against *S. aureus* strains with the same or lower MIC. Our model is based upon a single strain and with pharmacokinetic parameters derived from healthy animals. Rather it would be better to base our dose determination on the MIC_90_ values and to obtain more information about the population pharmacokinetics of cefquinome to take into account all the variability associate to animals and bacteria.

The therapeutic effect of an antimicrobial agent depends on several factors such as disease severity, animal immune response, pathogen load and strain virulence. The objective of the approach described here is to clear an organism of a pathogen, which was expected to be present in the central compartment during acute infection. Our model for *S. aureus* with a MIC of 0.25 μg/mL lead to AUC_24 h_/MIC for bacteriostasis and bactericidal action close 30 and 52 h which are higher than those (21 and 35 h, respectively) reported in piglet cage tissue fluid for a strain of *E. coli* with an MIC of 0.03 μg/mL (Zhang et al., 2014). The PK-PD surrogates have been widely used to provide dosages that aim to ensure clinical cures. These surrogates are less appropriate to prevent emergence of resistance strains and it has been suggested that the AUC/MPC ratio could serve as an indicator of drug exposure that prevents the selection of drug resistant mutant. However, this ratio should be a surrogate of the prevention of resistance selection at the target site. But, the same approaches must be applied to other body compartments such as the intestinal lumen where the microbiota can be exposed to selective concentration of the drug (Zhang et al., 2013).

Resistance to antimicrobials is a major threat for human health, the overuse and misuse is considered to be the main factor for increasing bacterial resistance in both humans and animals. The gut microbiota constitutes one of the key reservoirs of resistance genes between commensal bacterial ecosystems (Andremont, 2003; Phillips et al., 2004; Baquero et al., 2011; De Lastours et al., 2012; Vasseur et al., 2014). Antibiotic dosages presently used in humans and animals have not been developed to prevent the collateral choice on the gut microbiota and the selection and amplification of resistant strains (Fantin et al., 2009; De Lastours et al., 2012; Vasseur et al., 2014). An investigation of the interactions between microbial populations and antibiotics, as well as better understanding of the significant factors governing antimicrobial action and resistance range, might lead to the development of strategies combining maximum efficacy with minimum impact on the commensal bacterial ecosystems (Baquero et al., 2011; Cantón and Morosini, 2011; Martinez et al., 2012; Vasseur et al., 2014). For example, current studies confirmed that the degree of increasing of antimicrobial resistance in the gut microbiota was directly associated with the magnitude of the antibiotic dosage, irrespective of the route of administration (Nguyen et al., 2012; Zhang et al., 2013; Vasseur et al., 2014). Therefore there is an essential need to improve the antibiotic dose by taking this effect in the process of drug optimization.

## Conclusion

The purpose of our study was to estimate a dosage regimen of cefquinome after intravenous administration that would be efficient for treatment of *S. aureus* septicemia in cattle. The dosage estimate was based on a pharmacokinetic analysis and some pharmacodynamic studies. As previously described we observed that cefquinome is a time dependent drug with a low *in vitro* PAE for *S. aureus*. Pharmacodynamic parameters were derived from the analysis of static time kill curves obtained *ex-vivo*. The dosage regimen was simulated using a mathematical model. For a time dependent drug, this approach for dose determination is better than the analysis of the relationship between AUC/MIC value and the number of viable bacteria after 24 h. A dosage regimen of 2 mg/kg every 12 h during 3 days should be efficient in the treatment of *S. aureus* septicemia.

### Author contributions

Design of the work: HH, LH, ZY. Analysis and interpretation of the work: PS, IA. Drafting the work or revising it critically for important intellectual content: DC, YT, SX, XW. Experimental procedure performed: IA. Paper writing: IA, JL, WD, KX.

### Conflict of interest statement

The authors declare that the research was conducted in the absence of any commercial or financial relationships that could be construed as a potential conflict of interest.
